# Identification of pseudo-immune tolerance for chronic hepatitis B patients: Development and validation of a non-invasive prediction model

**DOI:** 10.3389/fpubh.2023.1137738

**Published:** 2023-04-05

**Authors:** Shuo Li, Zhiguo Li, Hongbo Du, Xiaobin Zao, Da’nan Gan, Xianzhao Yang, Xiaoke Li, Yufeng Xing, Yong’an Ye

**Affiliations:** ^1^Department of Gastroenterology, Dongzhimen Hospital, Beijing University of Chinese Medicine, Beijing, China; ^2^Institute of Liver Diseases, Beijing University of Chinese Medicine, Beijing, China; ^3^Department of Gastroenterology, Beijing Fengtai Hospital of Integrated Traditional and Western Medicine, Beijing, China; ^4^Department of Hepatology, Shenzhen Traditional Chinese Medicine Hospital, Shenzhen, China

**Keywords:** chronic hepatitis B, liver fibrosis, immune tolerant, liver biopsy, nomogram

## Abstract

**Background and aims:**

Patients with chronic hepatitis B (CHB) in the immune tolerant (IT) phase were previously thought to have no or slight inflammation or fibrosis in the liver. In fact, some CHB patients with normal ALT levels still experience liver fibrosis. This study aimed to develop and validate a non-invasive model for identifying pseudo-immune tolerance (pseudo-IT) of CHB by predicting significant liver fibrosis.

**Methods:**

This multi-center study enrolled a total of 445 IT-phase patients who had undergone liver biopsy for the training cohort (*n* = 289) and validation cohort (*n* = 156) during different time periods. A risk model (IT-3) for predicting significant liver fibrosis (Ishak score ≥ 3) was developed using high-risk factors which were identified using multivariate stepwise logistic regression. Next, an online dynamic nomogram was created for the clinical usage. The receiver operating characteristic (ROC) curve, net reclassification improvement and integrated discrimination improvement were used to assess the discrimination of the IT-3 model. Calibration curves were used to evaluate the models’ calibration. The clinical practicability of the model was evaluated using decision curve analysis and clinical impact curves.

**Results:**

8.8% (39 of 445) patients presented with significant liver fibrosis in this study. Aspartate aminotransferase (AST), hepatitis B e-antigen (HBeAg), and platelet (PLT) were included in the prediction model (IT-3). The IT-3 model showed good calibration and discrimination both in the training and validation cohorts (AUC = 0.888 and 0.833, respectively). The continuous NRI and IDI showed that the IT-3 model had better predictive accuracy than GPR, APRI, and FIB-4 (*p* < 0.001). Decision curve analysis and clinical impact curves were used to demonstrate the clinical usefulness. At a cut-off value of 106 points, the sensitivity and specificity were 91.7 and 70.2%, respectively.

**Conclusion:**

The IT-3 model proved an accurate non-invasive method in identifying pseudo-IT of CHB, which can help to formulate more appropriate treatment strategies.

## Introduction

Hepatitis B virus (HBV) infection is a serious public health problem worldwide which affects approximately 240 million individuals (1, 2). It is estimated that there are more than 50 million people in the immune tolerant (IT) phase. Previous studies (3–6) thought that IT-phase patients had slow disease progression due to little inflammation or fibrosis in liver. IT-phase patients still had poor rates of seroconversion after receiving antiviral therapy, and they were more likely to develop treatment resistance (7). Therefore, most international clinical guidelines (8–10) recommend that treatment in the IT phase be primarily based on regular monitoring instead of using nucleoside analogs or interferons. However, progression of the disease was observed in IT-phase patients during long-term follow-up, eventually resulting in cirrhosis, liver cancer, and other adverse outcomes (11). The definition of the IT phase was usually based on three main criteria: the serum HBV DNA level, the serum ALT level and the histological features of the liver. In fact, the levels of ALT were not fully representative of the extent of liver damage. Several studies (12, 13) showed that a proportion of HBeAg-positive patients with normal ALT levels actually had significant liver inflammation and fibrosis. The normal ALT levels were most likely just a false appearance of immune tolerance, as significant liver fibrosis suggested that immune responses had already occurred.

Additionally, the definition and management of IT-phase patients were not completely consistent in the clinical guidelines published by the EASL (8), AASLD (9), and APASL (10). The main differences were reflected in age, the ULN of ALT, and HBV DNA load. These differences made clinical stage and treatment ambiguous and might lead to inappropriate treatment for a certain group of patients. In order to provide accurate and individualized treatments, it was essential to identify pseudo-immune tolerance (pseudo-IT) patients from those with normal ALT. Due to the dynamic reciprocal process between immune tolerance and immune clearance, patients are at risk of developing liver fibrosis during the progression of CHB, even if they were previously diagnosed as immune tolerant. However, these patients were frequently neglected for treatment due to normal ALT levels. Histological evidence of liver is a breakthrough in identifying the pseudo-immune tolerance. Although liver biopsy was the gold standard for determining liver histology, it was impractical to use it on a regular basis because of its invasiveness. There is an urgent clinical need for a non-invasive diagnostic method to assess liver fibrosis in IT-phase patients.

In this study, we explored risk factors for liver fibrosis and developed a non-invasive nomogram model for identifying pseudo-IT of CHB from a large retrospective, biopsy-based, multi-center cohort study.

## Methods

### Study design

The patients were screened from 18 medical centers in different areas of China (Supplementary Table S1). We followed the TRIPOD guideline (14) (transparent reporting of a multivariable prediction model for individual prognosis or diagnosis) for training, validation and reporting of the proposed nomogram. This study was approved by the Ethics Committees of the Dongzhimen Hospital, Beijing University of Chinese Medicine. Written informed consent was provided by all patients.

### Patients

The following inclusion criteria were listed (8–10) (1) positive serum HBeAg; (2) HBsAg present for ≥6 months; (3) HBV DNA > 10^6^ IU/mL; (4) age > 18 years old; (5) persistently ALT <40 U/L at least 3 times in 12 months. Exclusion criteria included the following: (1) presence of other etiologies of liver diseases (e.g., viral coinfection, autoimmune hepatitis, alcoholic liver disease, nonalcoholic fatty liver disease); (2) taking antiviral drugs 6 months before enrollment; (3) liver cirrhosis or carcinoma; (4) patients with systemic diseases affecting the liver (e.g., HIV infection, heart failure, or thyroid).

**Figure 1:**
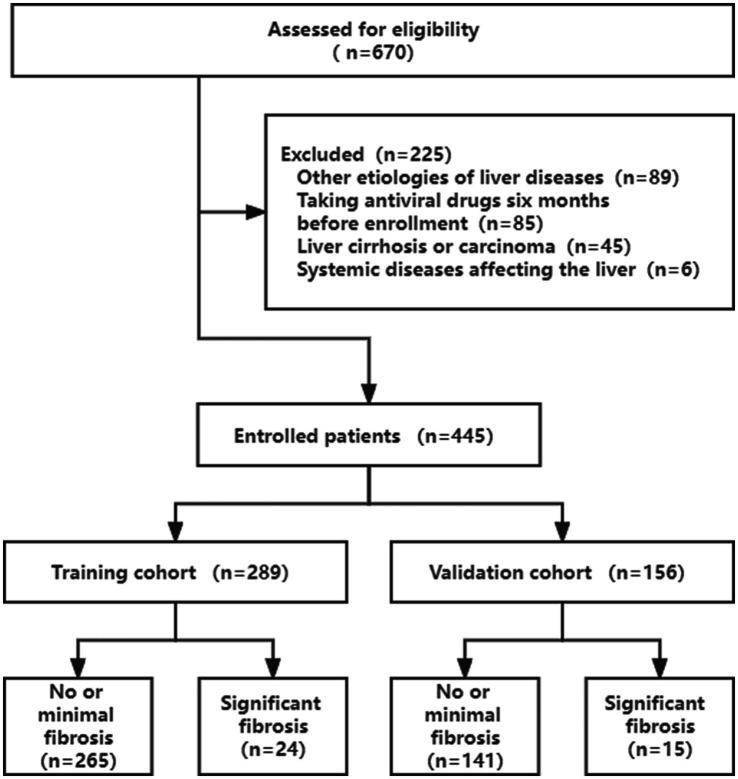
Flow chart presenting the study subjects.

A total of 670 eligible patients were retrospectively screened for this study. According to the exclusion criteria, 225 (33.6%) patients were excluded. 289 patients were in the training cohort (from May 2009 to May 2016), whereas the validation cohort included 156 patients (from May 2016 to May 2019) (Figure 1).

### Definition

Assessment of liver fibrosis using the Ishak’s system (15). The fibrosis stage was graded from stage 0–6. Stage 0–2 indicated no or minimal liver fibrosis, and stage 3–6 indicated significant liver fibrosis.

### Collection of clinical and pathological data

We collected baseline clinical and pathological data of 445 patients, including age, gender, body mass index (BMI), histological assessment, blood routine, hepatic and renal function, serological markers of HBV, and HBV DNA load from their electronic medical records.

The formula for calculating aspartate aminotransferase to platelet ratio index (APRI) (16), fibrosis index based on the four factors (FIB-4) (17) and gamma-glutamyl transpeptidase to platelet ratio (GPR) (18) was as previously described:


APRI=(AST/itsULN)/platelet count×100

$$\mathrm{FIB}-4=\left(\mathrm{age}\times \mathrm{AST}\right)/\left(\mathrm{platelet count}\times {\left(\mathrm{ALT}\right)}^{1/2}\right)$$

GPR=(GGT/itsULN)/platelet count×100

### Histological assessment

Ultrasound-guided percutaneous liver biopsies were performed in all enrolled patients using 16-G tru-cut biopsy needles (Menghini, Bard Company of America). Following formalin fixation and paraffin embedding, the samples were stained with hematoxylin–eosin and reticular fibers. Two experienced pathologists assessed the samples while concealing the clinical information of participants. The stage of fibrosis was determined using the Ishak fibrosis score (IFS) (15) and hepatic inflammation was assessed using the modified Ishak histologic activity index (HAI) (19).

### Statistical analyses

Data analyses were performed using SPSS (version 26.0, IBM, NY) and R (version 4.2.0, Vienna, Austria). A two-tailed *p* < 0.05 was considered statistically significant. Continuous variables were compared using the Student t-test (normal distribution) and Mann–Whitney *U* test (skewed distribution), which were presented as mean ± standard deviation and median (interquartile range, IQR), respectively. Categorical variables were presented as number (percentage) and compared by the chi-square test or Fisher’s exact test. The high-risk factors for significant fibrosis were determined through univariate and multivariate logistic regression. The variables with a value of *p* < 0.05 in univariate analysis were subsequently selected and entered into multivariable logistic regression with the backward stepwise method (threshold = 0.1).

The nomogram was constructed based on proportionally converting each regression coefficient in multivariate logistic regression to a 0-to-100-point scale by using the “regplot” package in R. The area under the receiver operating characteristic curves (AUC) were used to assess the discrimination of nomogram. The continuous net reclassification improvement (NRI) and integrated discrimination improvement (IDI) were computed in order to evaluate the improvement and applicability of the new model in reclassification. Confidence intervals for NRI and IDI were generated with the bootstrap method with 1,000 replications. The calibration curve was used to evaluate the predictive performance of the model. A 1000-time bootstrap resampling was used to assess the stability of the model. Decision curve analysis (DCA) and clinical impact curve (CIC) analysis were used to assess the clinical utility of the models.

## Results

### Baseline characteristics

As shown in Table 1, a total of 445 patients were enrolled in the current study. The median age of participants was 32 years (IQR = 30–37), and 62.9% (280 of 445) were male. All the patients were divided into two sets, with 289 patients (64.9%) assigned to the training cohort and 156 patients (35.1%) assigned to the validation cohort, according to different enrollment periods. Among them, 39 patients (8.8%) showed significant liver fibrosis (IFS score ≥ 3). All the baseline characteristics were not statistically different between the training and validation cohorts (*p* > 0.05).

**Table 1:** Baseline characteristics of patients in the training and validation cohorts.

Variable	All patients (*n* = 445)	Training cohort (*n* = 289)	Validation cohort (*n* = 156)	*p*
Age(years)^a^	32.0 (30.0, 37.0)	32.0 (30.0, 36.0)	32.0 (30.0, 37.8)	0.830
Male sex^b^	280 (62.9)	185 (64.0)	95 (60.9)	0.516
BMI (kg/m^2^)^a^	21.7 (20.1, 23.4)	21.7 (20.2, 23.5)	21.6 (19.7, 23.4)	0.444
WBC (10^12^/L)^a^	5.6 (5.0, 6.6)	5.6 (5.0, 6.5)	5.6 (5.0, 6.7)	0.862
PLT (10^9^/L)^a^	189.0 (159.5, 216.5)	185.0 (158.5, 218.5)	192.5 (161.2, 214.0)	0.694
ALT (U/L)^a^	27.0 (21.0, 35.0)	28.0 (22.0, 36.1)	26.1 (20.0, 32.6)	0.063
AST (U/L)^a^	24.0 (20.0, 29.6)	25.0 (20.0, 30.0)	23.0 (20.0, 28.0)	0.069
GGT (U/L)^a^	19.0 (14.0, 27.6)	19.3 (13.9, 28.7)	19.0 (14.0, 26.0)	0.498
BUN (mmol/L)^a^	4.9 (4.1, 5.9)	4.8 (4.1, 6.0)	4.9 (4.2, 5.9)	0.158
Cr (umol/L)^a^	75.2 (63.0, 86.0)	74.5 (62.1, 85.0)	76.0 (64.0, 87.7)	0.195
HBV-DNA (log_10_ IU/ml)^a^	8.3 (7.9, 8.7)	8.3 (7.9, 8.8)	8.2 (7.8, 8.7)	0.092
HBsAg (log_10_ IU/ml)^a^	4.8 (4.5, 5.0)	4.8 (4.5, 5.0)	4.8 (4.6, 5.0)	0.106
HBeAg (S/CO)^a^	1245.2 (1089.0, 1365.8)	1237.6 (1084.2, 1356.0)	1265.7 (1124.8, 1397.2)	0.064
HBcAb (S/CO)^a^	11.7 (10, 12.9)	11.8 (10.1, 13.0)	11.4 (9.8, 12.9)	0.270
GPR^a^	0.2 (0.2, 0.3)	0.2 (0.2, 0.3)	0.2 (0.2, 0.3)	0.225
APRI^a^	0.3 (0.2, 0.4)	0.3 (0.2, 0.5)	0.3 (0.2, 0.4)	0.052
FIB-4^a^	0.8 (0.7, 1.1)	0.8 (0.7, 1.1)	0.8 (0.6, 1.1)	0.090
IFS ≥3 points^b^^,^^c^	39 (8.8)	24 (8.3)	15 (9.6)	0.641
HAI ≥4 points^b^^,^^c^	157 (35.3)	97 (33.6)	60 (38.5)	0.302

ALT, alanine aminotransferase; AST, aspartate transaminase; APRI, aspartate aminotransferase-to-platelet ratio index; BMI, body mass index; BUN, blood urea nitrogen; Cr, creatinine; FIB-4, fibrosis index based on the four factors; GGT, gamma-glutamyltransferase; GPR, gamma-glutamyl transpeptidase to platelet ratio; HAI, histology activity index; HBcAb, anti-hepatitis B core antigen; HBeAg, hepatitis B e-antigen; HBsAg, hepatitis B surface antigen; IFS, Ishak fibrosis score; PLT, platelet; RBC, red blood cell; WBC, white blood cell.

a Data are presented as median (interquartile range, IQR), *p* values were estimated by Mann–Whitney *U* test.

b Data are shown as case number (percentage), *p* values were estimated by chisquare test.

c Defined when HAI ≥ 4 points as significant inflammation and IFS ≥ 3 points as significant fibrosis.

*p*, compared the training cohort with the validation cohort.

### Univariate and multivariate logistic regression analyses

Univariate and multivariate logistic regression analyses were performed to confirm the potential predictors in the training cohort (Table 2 and Supplementary Figure S1). Based on the results of stepwise regression, three predictors were finally identified: PLT (OR, 0.990; 95% CI, 0.980–1.001; *p* = 0.084), AST (OR, 1.084; 95% CI, 1.010–1.164; *p* = 0.025) and HBeAg (OR, 0.997; 95% CI, 0.996, 0.998; *p* < 0.001).

**Table 2:** Univariable and multivariable analysis in the training cohort.

	Univariable		Multivariable^a^	
	OR (95% CI)	*p* value	OR (95% CI)	*p* value
Age (years)	0.972 (0.901, 1.048)	0.972		
Male sex	0.933 (0.393, 2.213)	0.874		
BMI (kg/m^2^)	0.959 (0.837, 1.098)	0.542		
WBC(10^12^/L)	1.009 (0.875, 1.164)	0.900		
PLT(10^9^/L)	0.988 (0.978, 0.997)	0.013	0.990 (0.980, 1.001)	0.084
ALT(U/L)	1.086 (1.023, 1.153)	0.011	–	–
AST(U/L)	1.105 (1.039, 1.175)	0.002	1.084 (1.010, 1.164)	0.025
GGT(U/L)	1.037 (1.005, 1.071)	0.025	–	–
BUN(mmol/L)	0.900 (0.648, 1.249)	0.529		
Cr (umol/L)	0.987 (0.960, 1.014)	0.335		
HBV-DNA (log_10_ IU/mL)	0.659 (0.398, 1.093)	0.106		
HBsAg (log_10_ IU/mL)	0.271 (0.136, 0.540)	<0.001	–	–
HBeAg (S/CO)	0.997 (0.996, 0.998)	<0.001	0.997 (0.996, 0.998)	<0.001
HBcAb (S/CO)	1.002 (0.964, 1.042)	0.903		

CI, confidence interval; OR, odds ratio.

a Variables found to be significant (*p* < 0.05) by univariate analysis were entered into multivariate logistic regression analysis with backward stepwise method (threshold = 0.1).

### Nonivasive nomogram development

**Figure 2:**
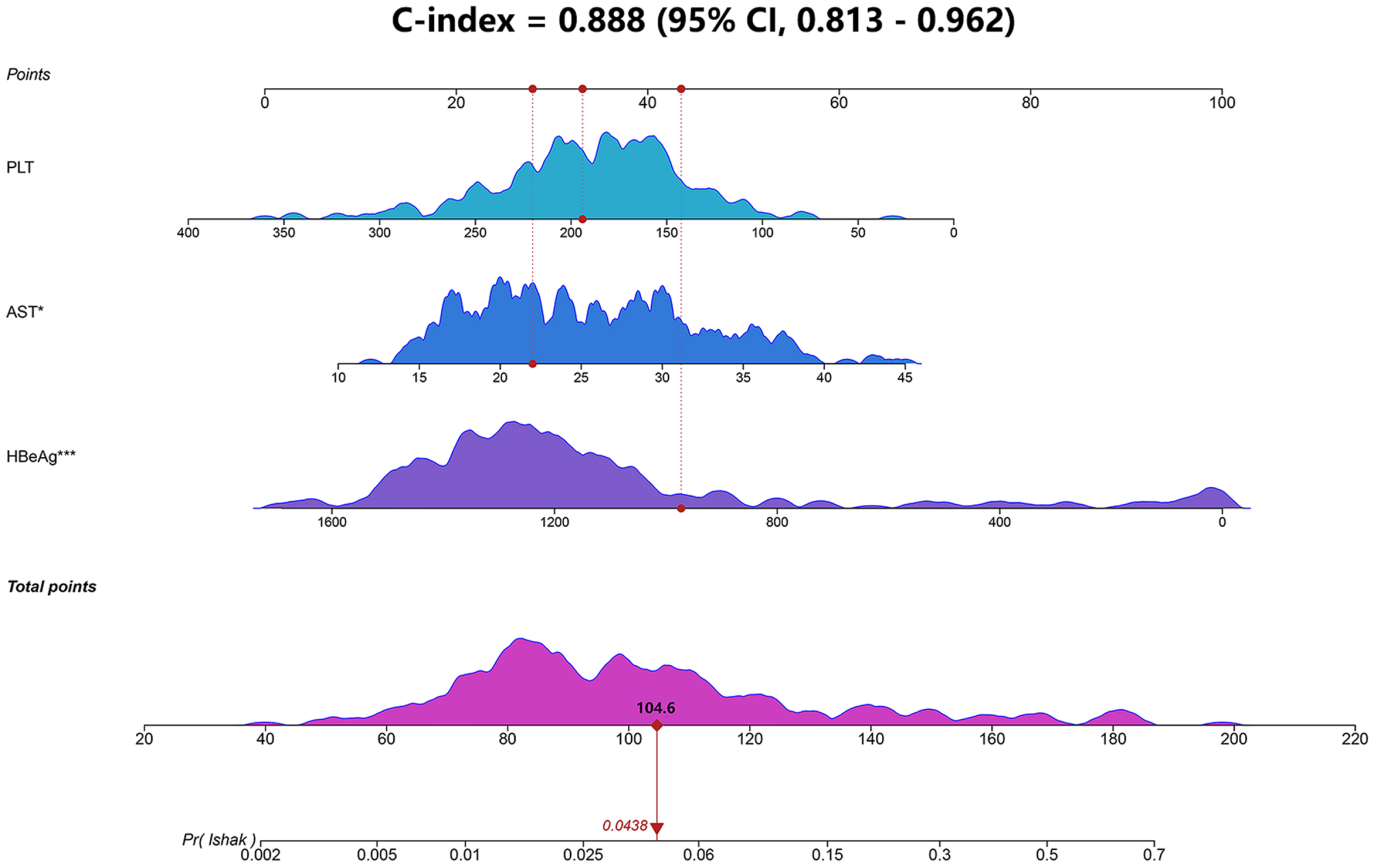
Nomogram (IT-3) for predicting liver fibrosis in IT-phase patients. The IT-3 model was developed using the training cohort and discrimination was evaluated by concordance index (Cindex). AST, aspartate transaminase; HBeAg, hepatitis B e-antigen; PLT, platelet.

Based on the logistic stepwise regression analysis, a nomogram was developed to predict the significant liver fibrosis for IT-phase patients and was named the IT-3 model (Figure 2). A total score was calculated by summing all predictors scores. The higher score suggests a higher risk of significant liver fibrosis. In addition, we created an online dynamic nomogram (Supplementary Figure S2).^1^

### IT-3 model evaluation

**Figure 3:**
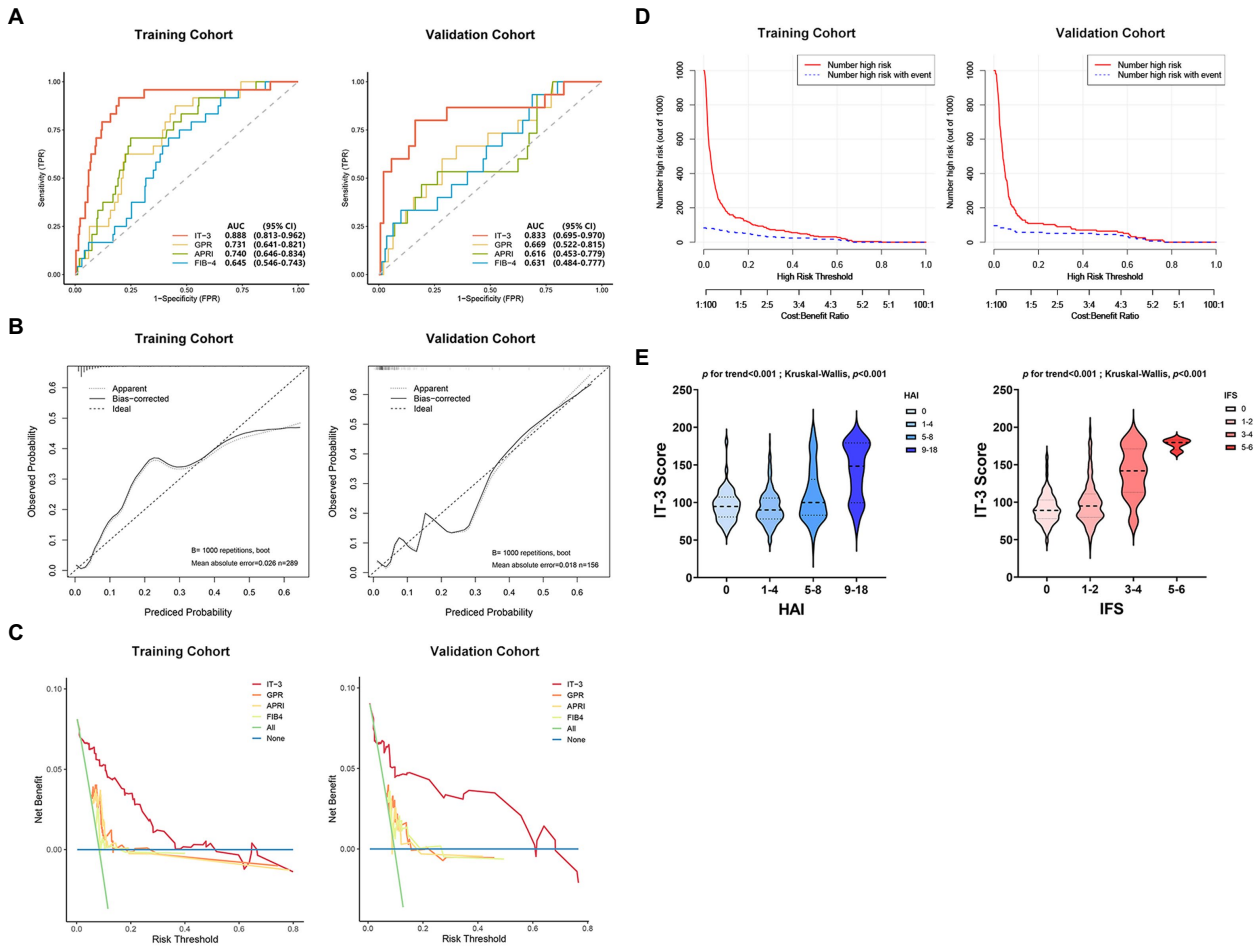
The IT-3 model performance evaluation. **(A)** Comparison of the area under the curve (AUC) between the IT-3 model and other nonivasive models. **(B)** Calibration curves. **(C)** Comparison of the decision curve analysis between the IT-3 model and other nonivasive models. **(D)** Clinical impact curves. **(E)** Relationship between IT-3 scores and liver pathology. APRI, aspartate aminotransferaseto-platelet ratio index; AUC, the area under the curve; FIB-4, fibrosis index based on the four factors; GPR, gamma-glutamyl transpeptidase to platelet ratio; HAI, histology activity index; IFS, Ishak fibrosis score.

We evaluated the IT-3 model through discrimination, calibration, and clinical decision benefit. In the training cohort, IT-3 had a higher AUROC [0.888 (0.813–0.962)] than GPR [0.731 (0.641–0.821), *p* = 0.007], APRI [0.74 (0.646–0.834), *p* = 0.001], and FIB-4 [0.645 (0.546–0.743), *p* < 0.001]. In the validation cohort, IT-3 had a higher AUROC [0.833 (0.695–0.970)] than GPR [0.731 (0.641–0.821), *p* = 0.147], APRI [0.616 (0.453–0.779), *p* = 0.009], and FIB-4 [0.631 (0.484–0.777), *p* = 0.050] (Table 3 and Figure 3A). The continuous NRI and IDI showed that the IT-3 model had better predictive accuracy than GPR, APRI, and FIB-4 (*p* < 0.001, Table 3). Using a cutoff value of 106 points, the sensitivity was 91.7% and the specificity was 70.2% in the training cohort. In the validation cohort, the sensitivity was 80.0%, and the specificity was 83.0%. The IT-3 model was validated in the 1,000-time bootstrap resampling with an AUC of 0.888 (95% CI 0.810–0.947) in the training cohort and 0.833 (95% CI 0.687–0.950) in the validation cohort. The IT-3 model also showed good accuracy after 1,000-time bootstrap resampling (Table 4).

**Table 3:** Discrimination of the IT-3 model and other non-invasive models.

	AUC (95%CI)	*p-*value^a^	NRI (95%CI)^b^	*p-*value	IDI (95%CI)^c^	*p-*value
Training cohort						
IT-3	0.888 (0.813–0.962)	–	–	–	–	–
GPR	0.731 (0.641–0.821)	0.007	1.27 (0.938–1.610)	<0.001	0.21 (0.124–0.302)	<0.001
APRI	0.740 (0.646–0.834)	0.001	1.36 (1.023–1.691)	<0.001	0.22 (0.134–0.302)	<0.001
FIB-4	0.645 (0.546–0.743)	<0.001	1.40 (1.086–1.704)	<0.001	0.23 (0.142–0.317)	<0.001
Validation cohort						
IT-3	0.833 (0.695–0.970)	–	–	–	–	–
GPR	0.669 (0.522–0.815)	0.147	1.21 (0.750–1.672)	<0.001	0.29 (0.145–0.433)	<0.001
APRI	0.616 (0.453–0.779)	0.009	0.97 (0.466–1.480)	<0.001	0.30 (0.153–0.441)	<0.001
FIB-4	0.631 (0.484–0.777)	0.050	1.00 (0.496–1.507)	<0.001	0.29 (0.149–0.426)	<0.001

APRI, aspartate aminotransferase-to-platelet ratio index; AUC, the area under curve; CI, confidence interval; FIB-4, fibrosis index based on the four factors; GPR, gamma-glutamyl transpeptidase to platelet ratio; IDI, integrated discrimination improvement; NRI, net reclassification improvement.

^a^ Compared with the IT-3 model.

^b,c^ NRI or IDI > 0 indicated the new model (IT-3) had better prediction performance than reference model (GPR, APRI or FIB-4). Cut-off of NRI: 0.2, 0.4.

**Table 4:** Performance and stability of the IT-3 model.

	Training cohort	Validation cohort
	(*n* = 289)	(*n* = 156)
Brier score	0.06	0.06
Sensitivity (%)	91.7	80.0
Specificity (%)	70.2	83.0
1,000-time bootstrap AUC (95% CI)	0.888 (0.810–0.947)	0.833 (0.687–0.950)
1,000-time bootstrap accuracy (%)	90.9	91.3

AUC, the area under curve; CI, confidence interval.

The calibration curve showed good agreement between the predicted and observed probabilities in the training and validation cohorts (brier score was 0.06 and 0.06, respectively) (Figure 3B and Table 3). The DCA of the IT-3 model demonstrated a greater net benefit with a wider range of threshold than the other non-invasive models in the training and validation cohorts (Figure 3C). The results of the clinical impact curves showed that the IT-3 model predictions had better agreement with the true positive rates. As the risk threshold increased, there was a decrease in unnecessary treatment and an increase in net clinical benefit (Figure 3D). The risk scores of patients were evaluated based on the IT-3 model were significantly correlated with the extent of liver inflammation or fibrosis (*p* < 0.001) (Figure 3E).

### Relationship between serological indicators and liver fibrosis and inflammation

**Figure 4:**
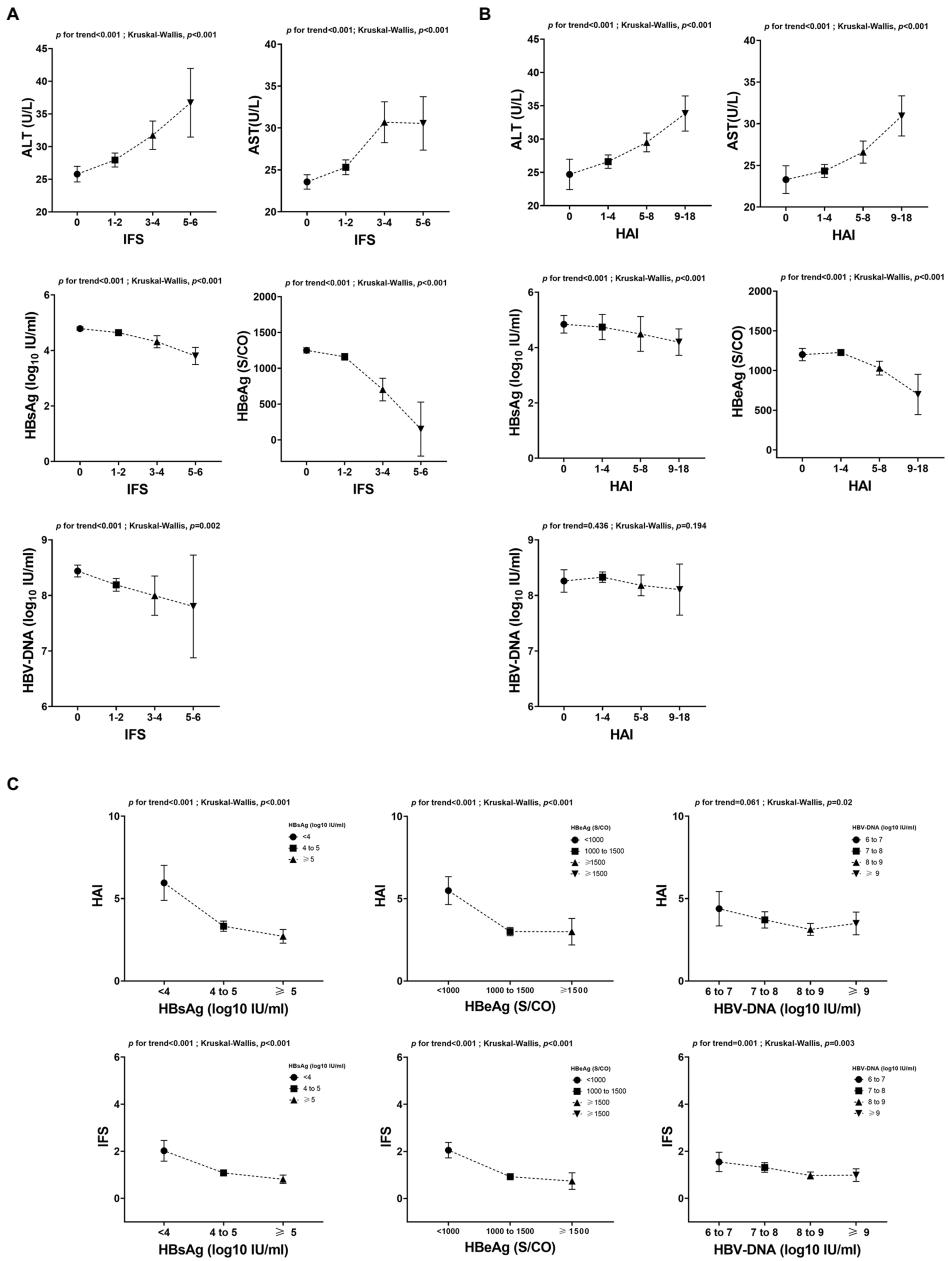
Relationship between serological indicators and liver pathology. **(A)** Relationship between serological indicators and the extent of fibrosis. **(B)** Relationship between serological indicators and the extent of inflammation. **(C)** Relationship between liver pathology and different levels of virological indicators. ALT, alanine aminotransferase; AST, aspartate transaminase; HAI, histology activity index; HBeAg, hepatitis B e-antigen; HBsAg, hepatitis B surface antigen.

According to the stage of liver fibrosis, patients were divided into different groups (IFS 0, 41.8%; IFS 1–2, 49.4%; IFS 3–4, 7.9%; IFS 5–6, 0.9%). A strong association was noted between serological indicators and the extent of fibrosis (Figure 4A). Significant fibrosis was associated with increasing levels of ALT (*p* for trend<0.001; K-W test *p* < 0.001) and AST (*p* for trend<0.001; K-W test *p* < 0.001), although the levels of transaminase were within the normal range. Significant fibrosis was associated with decreasing levels of HBsAg (*p* for trend <0.001; K-W test *p* < 0.001), HBeAg (*p* for trend <0.001; K-W test *p* < 0.001) and HBV-DNA (*p* for trend<0.001; K-W test *p* = 0.002). There was a similar trend when patients were grouped by liver inflammatory activity (HAI 0, 11.2%; HAI 1–4, 60.0%; HAI 5–8, 24.5%; HAI 9–18, 4.3%), although no statistically significant differences were observed in HBV DNA (Figure 4B). Then, patients were stratified according to different levels of virological indicators and found that both HAI and IFS tended to decrease as the virological indicators increased (*p* for trend <0.001; K-W test *p* < 0.001) (Figure 4C).

## Discussion

Due to the disease dynamics, it was important for IT-phase patients to monitor the liver histology in order to initiate antiviral treatments on time. In this study, we analyzed 445 IT-phase patients from 18 hospitals and developed a prediction model (IT-3) based on three non-invasive factors from a training cohort of 289 cases and validated in an external validation cohort of 156 cases. We found that lower HBeAg, higher AST, and lower PLT were high-risk factors for significant liver fibrosis in these patients. Base on the ROC, NRI and IDI analysis, the IT-3 model showed good prediction performance in predicting significant liver fibrosis and outperformed conventional models (APRI, GPR and FIB-4) in both training and validation cohorts. We demonstrated its good reliability and robustness by using advanced statistical methods (brier score and 1,000-time bootstrap validation). The risk scores calculated by the IT-3 model and the histology scores obtained from liver biopsies were in good agreement, indicating the ability of our model in assessing liver fibrosis. We also developed an online dynamic nomogram to make it easier to apply in clinical practice.

APRI (16), FIB-4 (17), and GPR (18) were non-invasive models commonly used for liver fibrosis assessment. However, we found that these ratio models did not show excellent performance in IT-phase patients. It might be attributed to the fact that the indicators used for prediction in the IT phase were almost entirely within the normal range, which limited the ability to assess of these ratio models. Therefore, the inclusion of virological indicators was necessary for liver fibrosis assessment in IT-phase patients. Several studies (20, 21) constructed non-invasive models to predict the risk of liver fibrosis for IT-phase patients, but the number of cases in the training cohorts was relatively small. Beyond this, external validation, model calibration, and decision curve analysis were not performed in these studies. Our model addressed these deficiencies and showed better discrimination. In comparison to the fibrosis staging diagnostic model developed by Wu et al. (22), our study also showed better discrimination, sensitivity and specificity in predicting significant liver fibrosis.

AST and HBeAg were independent predictors of liver fibrosis in IT-phase patients. ALT and AST were found in the cytoplasm and mitochondria, respectively. Thus, the rise in AST implied a deeper extent of liver injury and a greater likelihood of inflammatory infiltrates and desmoplasia, which might explain why AST, but not ALT, was an independent predictor in this study. Another important finding was that HBeAg levels were inversely correlated with the extent of liver fibrosis. HBeAg is an important indicator of viral replication and activity. However, when it was at a low level in IT-phase patients who were not receiving antiviral treatment, a possible explanation was the presence of immune-mediated viral clearance in the liver and it was the immunological reaction results in liver fibrosis. In fact, it was inaccurate to determine pathological status only based on the upper limit of normal (ULN) of transaminase. We observed that ALT and AST showed an increasing trend with increasing liver fibrosis, although the transaminases were within normal ranges. These findings suggested that it might be more beneficial for IT-phase patients to start antiviral therapy at a lower ULN, no longer using 40 U/L as the ULN for ALT, which was also consistent with some guidelines and opinions (9, 23). We also discovered that patients with significant fibrosis had lower levels of HBsAg and HBV DNA than patients with no or minor fibrosis, which was in line with previous studies (24–26) that found a negative correlation between these virological indicators and the stage of fibrosis in HBeAg-positive CHB patients.

There were some limitations to our study. Although this study was a multi-center study, the participants were all Chinese, and the majority of patients were of Asian ethnicity with genotypes B or C. The efficacy of this model for other races and genotypes remains to be validated. Second, the individuals in this study were all older than 18 years, which might limit the applicability in pediatric IT-phase patients. Third, we did not include transient elasography as a predictor variable when developing our model due to limited availability in China.

In conclusion, this study has developed a non-invasive and accurate model to predicting liver significant fibrosis for pseudo-immune tolerance patients and to provide more suitable therapeutic treatment regimens.

## Data availability statement

The raw data supporting the conclusions of this article will be made available by the authors, without undue reservation.

## Ethics statement

The studies involving human participants were reviewed and approved by the Dongzhimen Hospital affiliated to Beijing University of Chinese Medicine. The patients/participants provided their written informed consent to participate in this study. Written informed consent was obtained from the individual(s) for the publication of any potentially identifiable images or data included in this article.

## Author contributions

YY and YX: study concept and design. YX: acquisition of data. SL and ZL: analysis and interpretation of data and drafting of the manuscript. XL, HD, DG, XZ, and XY: critical revision of the manuscript for important intellectual content. XL and YY: study supervision. All authors read and approved the final version of the manuscript.

## Funding

This work was supported by grants from the National Major Science and Technology Projects of China (No. 2018ZX10725505), the National Natural Science Foundation of China (No. 82174341), and the Beijing University of Chinese Medicine Major Project (No. 2020-JYB-ZDGG-115).

## Conflict of interest

The authors declare that the research was conducted in the absence of any commercial or financial relationships that could be construed as a potential conflict of interest.

## Publisher’s note

All claims expressed in this article are solely those of the authors and do not necessarily represent those of their affiliated organizations, or those of the publisher, the editors and the reviewers. Any product that may be evaluated in this article, or claim that may be made by its manufacturer, is not guaranteed or endorsed by the publisher.

## Footnotes

^1^ https://nomogramit3.shinyapps.io/IT3model/
