# Supplementary material for: Identification of pseudo-immune tolerance for chronic hepatitis B patients: Development and validation of a non-invasive prediction model
Source: Front Public Health. 2023 Apr 5;11:1137738. doi: 10.3389/fpubh.2023.1137738 (PMC10113541; doi:10.3389/fpubh.2023.1137738)
Supplement: Supplementary file 1 [file Data_Sheet_1.pdf]

supplemental information

Table S1. Hospitals or medical centers that participated in this study.

Name
Shenzhen Traditional Chinese Medicine Hospital, Shenzhen, China
The Third People's Hospital of Shenzhen, Shenzhen, China
The Third Affiliated Hospital, Sun Yat-sen University, Guangzhou, China
The Traditional Chinese Medicine Hospital of Guangdong, Zhuhai Hospital, Zhuhai, China
The Traditional Chinese Medicine Hospital of Guangdong Province, Guangzhou, China
Foshan Traditional Chinese Medicine Hospital, Foshan, China
First Teaching Hospital of Tianjin University of Traditional Chinese Medicine, Tianjin, China
302 Military Hospital of China, Beijing, China
Beijing Ditan Hospital, Capital Medical University, Beijing, China
Xiyuan Hospital CACMS, Beijing, China
Beijing You An Hospital, Capital Medical University, Beijing, China
West China School of Medicine /West China Hospital, Sichuan University, Nanchong, China
Chengdu University of Traditional Chinese Medicine Hospital, Chengdu, China
Shuguang Hospital, Capital Shanghai University of Traditional Chinese, Shanghai, China
The Second Military Medical University of ChangHai Hospital, Shanghai, China
The Second Affiliated Hospital of Zhejiang Chinese Medical University, Hangzhou, China
Taian Traditional Chinese Medicine Hospital, Taian, China
Wuhan Medical and Treatment Center, Wuhan, China

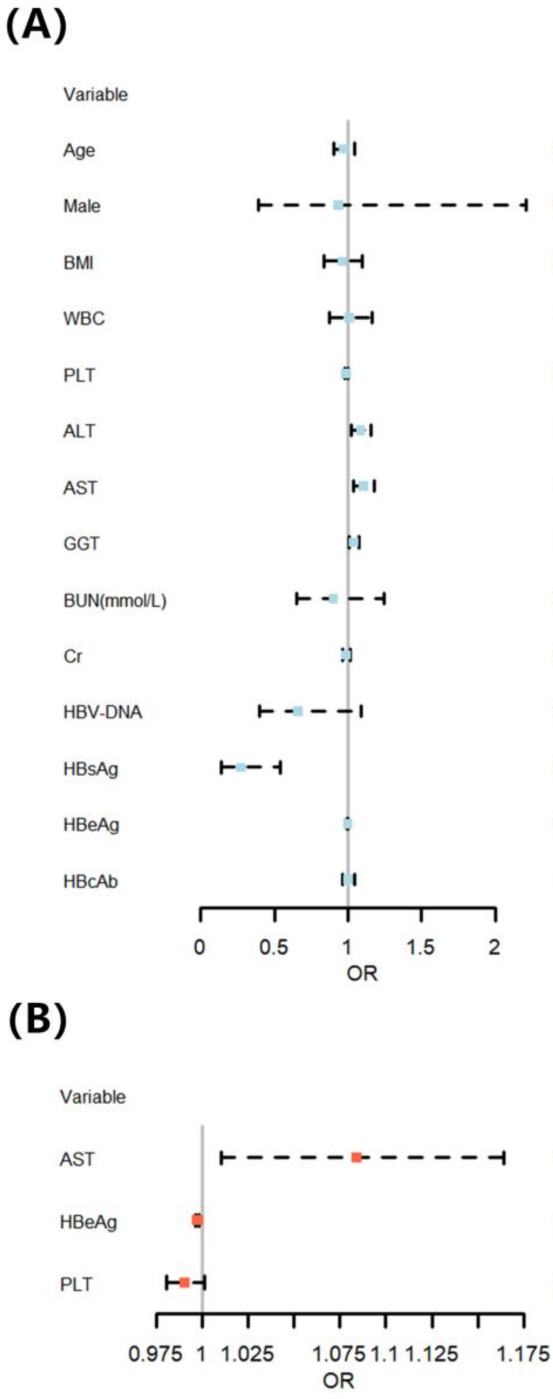

Fig.S1 Forest plots based on univariate (A) and multivariate (B) logistic regression analyses for predicting significant liver fibrosis in the training cohort. The solid box represents the OR and the dotted line shows 95% CI. ALT, alanine aminotransferase; AST, aspartate transaminase; BMI, body mass index; BUN, blood urea nitrogen; CI, confidence interval; Cr, creatinine; GGT, gamma-glutamyltransferase; HBcAb, anti-hepatitis B core antigen; HBeAg, hepatitis B e-antigen; HBsAg, hepatitis B surface antigen; OR, odds ratio; PLT, platelet; RBC, red blood cell; WBC, white blood cell.

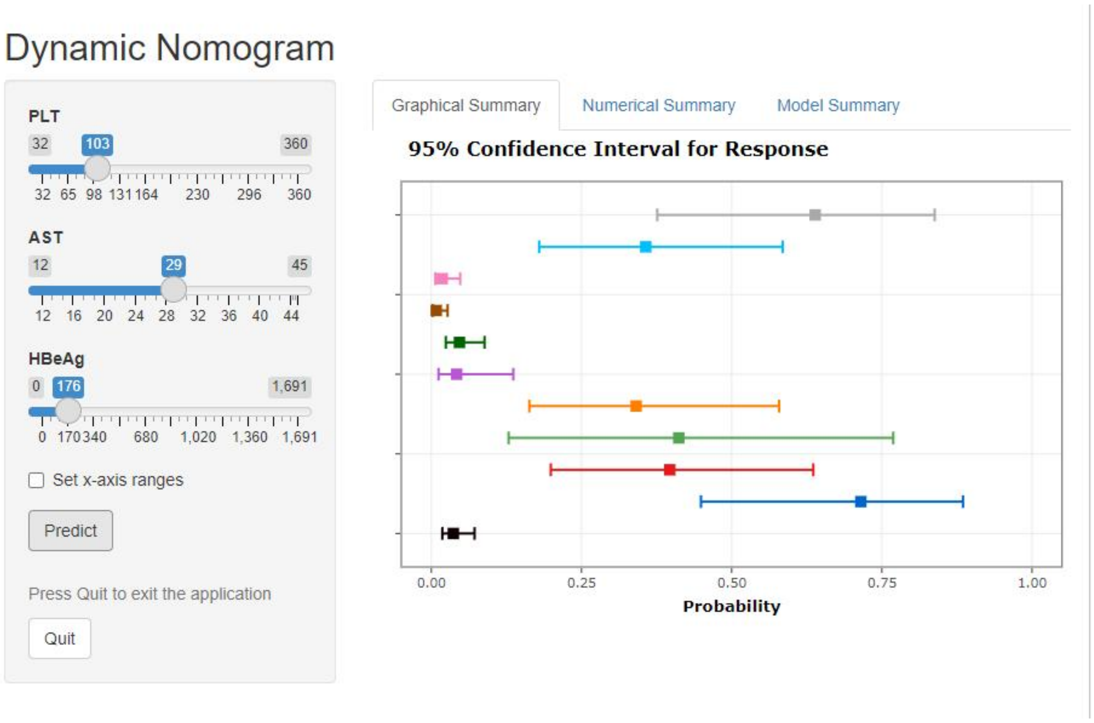

Fig. S2 The interface of the online dynamic nomogram. 95% confidence interval for probability.
